# Alpha-Amylase Activity in Blood Increases after Pharmacological, But Not Psychological, Activation of the Adrenergic System

**DOI:** 10.1371/journal.pone.0130449

**Published:** 2015-06-25

**Authors:** Urs M. Nater, Roberto La Marca, Katja Erni, Ulrike Ehlert

**Affiliations:** 1 Department of Psychology, University of Marburg, Marburg, Germany; 2 Department of Psychology, University of Zurich, Zurich, Switzerland; University College London, UNITED KINGDOM

## Abstract

**Background & Aim:**

Alpha-amylase in both blood and saliva has been used as a diagnostic parameter. While studies examining alpha-amylase activity in saliva have shown that it is sensitive to physiological and psychological challenge of the adrenergic system, no challenge studies have attempted to elucidate the role of the adrenergic system in alpha-amylase activity in blood. We set out to examine the impact of psychological and pharmacological challenge on alpha-amylase in blood in two separate studies.

**Methods:**

In study 1, healthy subjects were examined in a placebo-controlled, double-blind paradigm using yohimbine, an alpha2-adrenergic antagonist. In study 2, subjects were examined in a standardized rest-controlled psychosocial stress protocol. Alpha-amylase activity in blood was repeatedly measured in both studies.

**Results:**

Results of study 1 showed that alpha-amylase in blood is subject to stronger increases after injection of yohimbine compared to placebo. In study 2, results showed that there was no significant effect of psychological stress compared to rest.

**Conclusions:**

Alpha-amylase in blood increases after pharmacological activation of the adrenergic pathways suggesting that sympathetic receptors are responsible for these changes. Psychological stress, however, does not seem to have an impact on alpha-amylase in blood. Our findings provide insight into the mechanisms underlying activity changes in alpha-amylase in blood in healthy individuals.

## Introduction

Alpha-amylase is an enzyme that hydrolyzes internal alpha-1,4 glycoside bonds, resulting in the production of maltose and oligosaccharides. Amylase concentrations are highest in the pancreas and the salivary glands, although amylase is abundant in other organs as well. The salivary and pancreatic forms of alpha-amylase have gained interest in a variety of areas [1]. Measurement of salivary alpha-amylase activity has been proposed to reflect stress-related changes in the adrenergic system [2]. Studies using physiological [3, 4] and psychological stressors [5, 6] revealed increased activity of salivary alpha-amylase due to stress. Studies in humans have shown that alpha2-adrenergic [7] and beta-adrenergic [8] mechanisms are involved in increases of salivary alpha-amylase activity. Studies from our work group show that amylase activity in saliva is highly sensitive to psychological stressors and might thus be considered an easily measured non-invasive parameter to evaluate stress responses [9, 10]. On the other hand, measurement of alpha-amylase in blood has been used for the confirmation of acute pancreatitis and disease activity in a number of other conditions. Elevation of amylase levels in serum is a major indicator for acute pancreatitis, though controversial regarding its specificity [11–15].

Whereas salivary amylase is produced by the salivary glands and is released predominantly upon adrenergic innervation, amylase in blood is mostly produced and released by the pancreas. Pancreatic amylase enters the blood stream and can easily be measured in blood [16]. Pancreatic amylase was found in serum after beta-adrenergic stimulation [17]. However, unexplained elevations in serum alpha-amylase have caused puzzlement with regard to the etiology of these changes [18, 19].

Taken together, alpha-amylase in both blood and saliva has been used as a diagnostic parameter. Although amylase in serum is often measured as an indicator for acute pancreatitis, debates about its validity are found in the literature. Furthermore, since the salivary component of the enzyme has found to be subject to stress-related changes, one has to consider possible confounding values also in blood amylase due to stressful events, particularly given the involvement of the adrenergic system in the release of pancreatic amylase. We therefore set out to measure alpha-amylase activity in the blood in laboratory settings in which subjects were exposed to 1) a pharmacological and 2) a psychological challenge, both being believed to activate the adrenergic system, in two independent studies. We hypothesized that both a pharmacological and a psychological stimulation of the adrenergic system would elicit significant increases in alpha-amylase in blood.

## Methods

### Participants

In the first study (pharmacological challenge), a total of 13 healthy male subjects took part in the experiment. In the second study (psychological challenge), 32 healthy male subjects (not identical with subjects from the first study) participated. The following applies to participants of both studies:

All participants were recruited at the University of Zurich and the Swiss Federal Institute of Technology, Zurich, by electronic mail and/or advertisement. They received a screening questionnaire, containing exclusion criteria designed to reduce confounding factors that have been shown to affect physiological dependent variables. All subjects were medication-free. Furthermore, subjects were non-smokers, since smoking has been shown to increase serum amylase activity [20]. Participants were told not to perform any strenuous physical activity 48 hours prior to the experiment and to cease all sporting activities during the time of the study. Intake of ethanol and caffeine was forbidden 18 hours prior to the experiments. At least 60 minutes before the examinations, subjects were not allowed to eat and to take low pH soft drinks. Additionally, all subjects underwent a full health examination by a physician (study 1) or were screened for any health problems by a member of the study group (study 2). Subjects with any acute or chronic somatic or psychiatric disorder were excluded. After the subjects were provided with complete written and oral descriptions of the respective study, written informed consent was obtained. In both studies, the subjects were remunerated for participation. The study protocols were formally approved by the ethics committee of the University of Zurich and the ethics committee of the Canton of Zurich.

### Procedures

#### Pharmacological challenge

The effect of the alpha2-adrenergic receptor antagonist yohimbine on alpha-amylase activity in blood was determined in a randomized double-blind placebo controlled study. Fourty-five minutes prior to intravenous injection, a catheter line was inserted, which was kept patent by infusion of saline. Two samples were drawn before the injection (-20 and -10 minutes) of yohimbine hydrochloride (0.4 mg/kg) or 0.9% NaCl, respectively, and six additional samples were collected following the injection (+10, +20, +30, +60, +90, and +120 minutes). Alpha-amylase was determined from these samples (see below). The two conditions took place with a minimum of 14 days in between. All procedures were performed between 1300hours and 1700hours. Subjects were randomized into two groups, with group 1 undergoing first the placebo condition and group two undergoing first the yohimbine condition, to control for possible sequence effects between the two conditions.

#### Psychological challenge

A standardized psychosocial stress protocol was used. The Trier Social Stress Test (TSST) has repeatedly been found to induce profound psychological and physiological responses in 70–80% of the subjects tested [21]. Subjects were introduced to the TSST (two minutes). Afterwards, they had ten minutes to prepare their free speech, before subjects were exposed to a simulated job interview (five minutes) followed by a mental arithmetic task (five minutes) in front of an audience. Blood samples (by the indwelling venous catheter) were taken immediately before and after the TSST, with further samples taken at 15, 30, and 60 minutes to assess alpha-amylase. The stress and rest conditions were completed with a minimum of 14 days in between. The TSST was performed between 1300hours and 1700hours. Subjects were randomized into two groups, with group 1 undergoing first the rest condition and group two undergoing first the stress condition, to control for possible sequence effects between the two conditions.

### Measures

Blood was collected in EDTA-coated monovette (Sarstedt, Sevelen/Switzerland) collection devices. After centrifugation at 3000 rpm for 10 min, plasma was aliquoted in pre-cooled plastic tubes. Plasma amylase has been shown to be highly correlated to serum amylase [22, 23]. Samples were stored at –80°C until biochemical analysis took place. After thawing, samples were centrifuged again at 3000 rpm for 5 minutes. Amylase activity was expressed as enzyme level per volume of blood (enzymatic units per liter, U/l). Alpha-amylase activity was determined by using an automatic analyser (Cobas Mira) and assay kits obtained from Roche. The reagents in the kit contain the substrate 2-chloro-4-nitrophenyl maltotrioside which is hydrolysed by alpha-amylase and directly produces 2-chloro-4-nitrophenol (CNP). The rate of production of 2-chloro-4-nitrophenol, measured by the variation in absorbance per minute, is proportional to the alpha-amylase activity. The activity is determined by measuring the absorbance at 405nm. The assay is a kinetic colorimetric test. Inter- and intra-assay variance was below 1% in analyses of all of the experiments described in this paper.

### Statistical analyses

Differences between conditions were calculated with general lineal models and Student’s t-tests. ANOVAs for repeated measures were computed to reveal possible time and condition effects. All reported results were corrected by the Greenhouse-Geisser procedure where appropriate (violation of sphericity assumption). For alpha-amylase, area under the total response curve (AUC Total), expressed as area under all samples, as well as area under the response curve with respect to increase (AUC Increase), was calculated using the trapezoid formula [24]. Data were tested for normal distribution and homogeneity of variance using a Kolmogorov-Smirnov and Levene´s test before statistical procedures were applied. Effect sizes for repeated ANOVA (f^2^) were computed by the following formula: eta^2^ / 1—eta^2^. Effect sizes for group differences (d) were computed as the difference between the means, M_1_—M_2_, divided by standard deviation of either group. For all analyses, significance level was α = 5%. Unless indicated, all results shown are means ± standard error of means (SEM).

## Results

### Sample characteristics

In the first study, thirteen healthy male subjects participated (subject characteristics: age mean = 24.9 years, SD = 2.2 years; height mean = 180.0 cm, SD = 5.4 cm; weight mean = 73.5 kg, SD = 7.1 kg; BMI mean = 22.66, SD = 1.83).

In the second study, thirty-two healthy male subjects participated (subject characteristics: age mean = 23.8 years, SD = 2.4 years; height mean = 182.27 cm, SD = 7.66 cm; weight mean = 74.8 kg, SD = 9.22 kg; BMI mean = 22.48, SD = 1.98). Participation in the two studies was mutually exclusive.

### Pharmacological challenge

The administration of yohimbine resulted in significant changes of alpha-amylase over time (F (2.12/25.49) = 10.27; p < 0.001; f^2^ = 0.86; 95% CI 39.9–65.58 U/l). In the placebo condition, significant changes were also observed (F (3.58/42.99) = 4.22; p = 0.007; f^2^ = 0.35; 95% CI 41.32–66.72 U/l). Neither at –20 min (t12 = 1.55; p = 0.148), nor at +120 min (t12 = 0.25; p = .804) differences in amylase activity occurred between the two conditions. There was no condition main effect (F (1.00/12.00) = 0.54; p = 0.479; f^2^ = 0.04). However, both the main effect of time (F (2.49/29.85) = 12.20; p < 0.001; f^2^ = 1.02) and the interaction of condition x time were significant (F (2.64/31.68) = 5.54; p = 0.005; f^2^ = 0.46).

**Fig 1:**
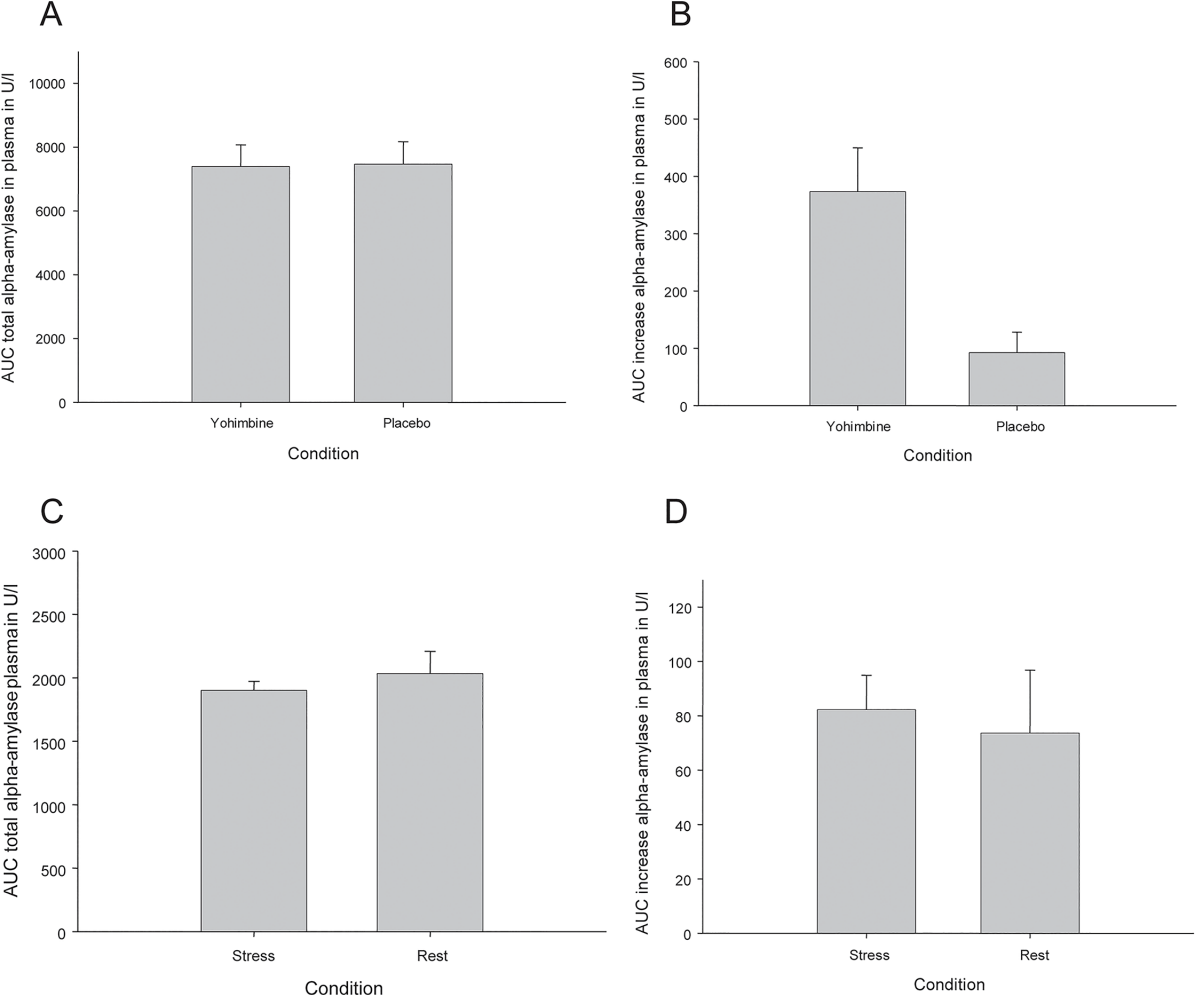
Alpha-amylase activity during four experimental conditions: pharmacological challenge and placebo (A AUC total and B AUC increase), and psychological challenge and rest (C AUC total and D AUC increase). Values are means (error bars indicate SEM).

The AUC Total of the two conditions did not differ significantly (t12 = 0.52; p = 0.610; 7549.96 U/l versus 7442.62 U/l; d = 0.04; 95% CI -339.16–553.85 U/l; Fig 1A). However, in the AUC Increase, a significant difference occurred, with the yohimbine condition resulting in a higher increase (t12 = -3.00; p = 0.011; 104.115 U/l versus 380.15 U/l; d = 0.93; 95% CI -476.7-(-)75.38 U/l; Fig 1B).

### Psychological challenge

The psychological stress test resulted in significant changes of alpha-amylase over time (F (2.73/79.26) = 46.86; p < 0.001; f^2^ = 1.62; 95% CI 47.7–62.76 U/l). In the rest condition, significant changes were also observed (F (1.62/48.61) = 12.66; p < 0.001; f^2^ = 0.42; 95% CI 46.65–71.04 U/l). Neither at the beginning (t28 = -1.16; p = 0.25), nor at the end (t28 = -1.39; p = 0.18) of the stress protocol differences in amylase activity occurred between the two conditions. There was no condition main effect (F (1.00/29.00) = 1.08; p = 0.31; f^2^ = 0.04), but a main effect of time (F (1.75/50.77) = 38.28; p < 0.001; f^2^ = 1.32). Furthermore, the interaction of condition x time was significant (F (2.52/72.93) = 4.31; p = 0.011; f^2^ = 0.15).

However, the AUC Total of the two conditions did not differ significantly (t29 = -1.03; p = 0.31; 1900.03 U/l versus 2062.17 U/l; d = 0.17; 95% CI -483.52–159.23 U/l; Fig 1C), and neither did the AUC Increase (t29 = 0.32; p = 0.75; 82.24 U/l versus 75.33 U/l; d = 0.01; 95% CI -36.91–50.73 U/l; Fig 1D).

## Discussion

We found marked increases in alpha-amylase in blood after challenge with a sympathetic stimulant (study 1), but not after a psychological stressor (study 2). Although significant time effects were observed in both the placebo condition of study 1 and in the rest condition of study 2, the effect sizes of the interventions (f^2^ yohimbine: 0.86, TSST: 1.62) markedly differed from the effect sizes of the respective control conditions (f^2^ placebo: 0.35, rest: 0.42). These results indicate that alpha-amylase in blood is subject to changes that are regulated by the adrenergic system.

The present findings may be compared to our findings in salivary amylase activity after challenge with a sympathetic stimulant [7] and psychological stress [9, 10]. This is interesting, since several studies have shown that parotid isoamylase is a component of circulating amylase (in addition to amylases originating from the pancreas and the liver also contributing to the sum of enzyme activity in blood). In a recent study, it was shown that pilocarpine administration-induced parotid amylase discharge in rats was accompanied by an increase in serum isoamylase levels [25]. The elevations found in our pharmacological study might be in part due to an increase of alpha-amylase in the salivary glands. Overall, pancreatic isoamylase represents around 40% of normal serum amylase activity [26]. With the analysis strategies used in the current studies, no information can be obtained on the exact contribution of the pancreas. Further studies should provide more in-depths analyses of specific amylase isoenzymes.

Alpha-amylase in blood has been studied as a diagnostic marker for differential pathological processes. As discussed, it is most often used to determine acute pancreatitis [11–15]. But several studies have used the enzyme in other conditions. In one study, e.g., total serum amylase was determined in patients with cystic fibrosis (CF), control patients, and patients with Shwachman's syndrome. The results demonstrate that the patients with Shwachman's syndrome had significantly lower total serum amylase than the other patient groups [27]. Also, hyperamylasemia and parotid hypertrophy are conditions found in patients with eating disorders [28–30]. One study examined serum amylase levels in 56 underweight anorectics, 24 weight-recovered anorectics, 23 normal-weight bulimics, and 31 volunteer women. Normal-weight bulimic patients had significantly higher admission serum amylase values than controls. The authors observed that modest increases of serum amylase values appear to be a consequence of binge-vomit behavior and suggested that serial serum amylase determination may be useful in monitoring the degree of patient abstinence in therapeutic programs [28]. In the serum and saliva of 45 patients with eating disorders and in 30 normal controls, alpha-amylase activity was measured. Of the 45 patients evaluated, 12 had restrictive anorexia nervosa, 13 were bulimic anorectics and 20 had bulimia nervosa. In all these groups, the mean alpha-amylase values in serum and saliva were higher than that of the control group. In all patients with eating disorders, the mean concentration and secretion of alpha-amylase in saliva were increased [29]. Another study investigated the clinical relevance of amylase level monitoring as an objective measure in diagnosis and assessment of treatment response in bulimia nervosa. Thirty-three subjects with bulimia nervosa had serum levels of total and salivary amylase monitored during an 8-week treatment trial. At the beginning of treatment, the average total amylase level was within the upper limits of normal, whereas average salivary amylase levels were abnormally high. During the course of treatment, there was a significant reduction in the average salivary isoenzyme to within the normal range. Significant reductions in amylase levels were recorded in patients with good treatment outcome, but not in those with poor outcome. Amylase levels were not significantly correlated with severity of bulimic symptoms. These results do not justify the use of amylase assays as a routine diagnostic or monitoring test, but isoenzyme monitoring may provide useful clinical information in selected cases [30]. Eating disorders are not the only pathological conditions related to changes in alpha-amylase levels. Both pancreatic and salivary isoenzymes in serum were examined in a study with 12 Alzheimer patients looking for the possibility of amylase as a marker of M3 activity associated with maximally tolerated dose of xanomeline tartrate. Overall amylase results were not significant; however, a trend for salivary amylase was found [31]. Taken together, alpha-amylase assessed in blood has been used widely as a diagnostic marker in various conditions; however, the mechanisms that underlying potential changes as well as the differential contribution of various sources of amylase are still not well understood and require further research efforts.

In our study, we were able to show that alpha-amylase in blood increases after induction of pharmacological activation of the adrenergic pathways. Further, since there were no discernable effects of psychological stress, it might be suggested that alpha-amylase levels in blood determined in routine and emergency analyses are most likely not confounded by everyday stressors or the small stress elicited by a venous puncture.

## References

[1] Zakowski JJ, Bruns DE. Biochemistry of human alpha amylase isoenzymes. Crit Rev Clin Lab Sci. 1985;21(4):283–322. . 2578342 10.3109/10408368509165786

[2] Nater UM, Rohleder N. Salivary alpha-amylase as a non-invasive biomarker for the sympathetic nervous system: current state of research. Psychoneuroendocrinology. 2009;34(4):486–96. doi: 10.1016/j.psyneuen.2009.01.014 19249160 10.1016/j.psyneuen.2009.01.014

[3] Nexo E, Hansen MR, Konradsen L. Human salivary epidermal growth factor, haptocorrin and amylase before and after prolonged exercise. Scand J Clin Lab Invest. 1988;48(3):269–73. . 2453916 10.3109/00365518809167494

[4] Walsh NP, Blannin AK, Clark AM, Cook L, Robson PJ, Gleeson M. The effects of high-intensity intermittent exercise on saliva IgA, total protein and alpha-amylase. J Sports Sci. 1999;17(2):129–34. . 10069269 10.1080/026404199366226

[5] Bosch JA, De Geus EJ, Veerman EC, Hoogstraten J, Nieuw Amerongen AV. Innate secretory immunity in response to laboratory stressors that evoke distinct patterns of cardiac autonomic activity. Psychosom Med. 2003;65(2):245–58. . 12651992 10.1097/01.psy.0000058376.50240.2d

[6] Chatterton RT Jr., Vogelsong KM, Lu YC, Hudgens GA. Hormonal responses to psychological stress in men preparing for skydiving. J Clin Endocrinol Metab. 1997;82(8):2503–9. . 9253325 10.1210/jcem.82.8.4133

[7] Ehlert U, Erni K, Hebisch G, Nater U. Salivary alpha-amylase levels after yohimbine challenge in healthy men. J Clin Endocrinol Metab. 2006;91(12):5130–3. . 16968802 10.1210/jc.2006-0461

[8] van Stegeren A, Rohleder N, Everaerd W, Wolf OT. Salivary alpha amylase as marker for adrenergic activity during stress: effect of betablockade. Psychoneuroendocrinology. 2006;31(1):137–41. . 16046076 10.1016/j.psyneuen.2005.05.012

[9] Nater UM, Rohleder N, Gaab J, Berger S, Jud A, Kirschbaum C, et al Human salivary alpha-amylase reactivity in a psychosocial stress paradigm. Int J Psychophysiol. 2005;55(3):333–42. . 15708646 10.1016/j.ijpsycho.2004.09.009

[10] Nater UM, La Marca R, Florin L, Moses A, Langhans W, Koller MM, et al Stress-induced changes in human salivary alpha-amylase activity—associations with adrenergic activity. Psychoneuroendocrinology. 2006;31(1):49–58. . 16002223 10.1016/j.psyneuen.2005.05.010

[11] Benifla M, Weizman Z. Acute pancreatitis in childhood: analysis of literature data. J Clin Gastroenterol. 2003;37(2):169–72. . 12869890 10.1097/00004836-200308000-00015

[12] Yadav D, Agarwal N, Pitchumoni CS. A critical evaluation of laboratory tests in acute pancreatitis. Am J Gastroenterol. 2002;97(6):1309–18. . 12094843 10.1111/j.1572-0241.2002.05766.x

[13] Smotkin J, Tenner S. Laboratory diagnostic tests in acute pancreatitis. J Clin Gastroenterol. 2002;34(4):459–62. . 11907364 10.1097/00004836-200204000-00018

[14] Vissers RJ, Abu-Laban RB, McHugh DF. Amylase and lipase in the emergency department evaluation of acute pancreatitis. J Emerg Med. 1999;17(6):1027–37. . 10595892 10.1016/s0736-4679(99)00136-5

[15] Lankisch PG, Burchard-Reckert S, Lehnick D. Underestimation of acute pancreatitis: patients with only a small increase in amylase/lipase levels can also have or develop severe acute pancreatitis. Gut. 1999;44(4):542–4. . 10075962 10.1136/gut.44.4.542 PMC1727444

[16] Pieper-Bigelow C, Strocchi A, Levitt MD. Where does serum amylase come from and where does it go? Gastroenterol Clin North Am. 1990;19(4):793–810. . 1702756

[17] Skov Olsen P, Kirkegaard P, Rasmussen T, Magid E, Poulsen SS, Nexo E. Adrenergic effects on secretion of amylase from the rat salivary glands. Digestion. 1988;41(1):34–8. . 2462520 10.1159/000199729

[18] Warshaw AL, Hawboldt MM. Puzzling persistent hyperamylasemia, probably neither pancreatic nor pathologic. Am J Surg. 1988;155(3):453–6. . 2449825 10.1016/s0002-9610(88)80112-0

[19] Levitt MD, Ellis CJ, Meier PB. Extrapancreatic origin of chronic unexplained hyperamylasemia. N Engl J Med. 1980;302(12):670–1. . 6153453 10.1056/NEJM198003203021206

[20] Dubick MA, Conteas CN, Billy HT, Majumdar AP, Geokas MC. Raised serum concentrations of pancreatic enzymes in cigarette smokers. Gut. 1987;28(3):330–5. . 2436981 10.1136/gut.28.3.330 PMC1432685

[21] Kirschbaum C, Pirke KM, Hellhammer DH. The 'Trier Social Stress Test'—a tool for investigating psychobiological stress responses in a laboratory setting. Neuropsychobiology. 1993;28(1–2):76–81. . 8255414 10.1159/000119004

[22] Gillard BK, Simbala JA, Goodglick L. Reference intervals for amylase isoenzymes in serum and plasma of infants and children. Clin Chem. 1983;29(6):1119–23. . 6189641

[23] Medaille C, Briend-Marchal A. Comparison of amylase and lipase activities in serum and plasma of dogs. Vet Clin Pathol. 2004;33(3):155–8. . 15334351 10.1111/j.1939-165x.2004.tb00366.x

[24] Pruessner JC, Kirschbaum C, Meinlschmidt G, Hellhammer DH. Two formulas for computation of the area under the curve represent measures of total hormone concentration versus time-dependent change. Psychoneuroendocrinology. 2003;28(7):916–31. . 12892658 10.1016/s0306-4530(02)00108-7

[25] Nagy A, Barta A, Varga G, Zelles T. Changes of salivary amylase in serum and parotid gland during pharmacological and physiological stimulation. J Physiol Paris. 2001;95(1–6):141–5. . 11595428 10.1016/s0928-4257(01)00018-3

[26] Ellis C, Koehler DF, Eckfeldt JH, Levitt MD. Evaluation of an inhibitor assay to determine serum isoamylase distribution. Dig Dis Sci. 1982;27(10):897–901. . 6180877 10.1007/BF01316573

[27] Davidson GP, Koheil A, Forstner GG. Salivary amylase in cystic fibrosis: a marker of disordered autonomic function. Pediatr Res. 1978;12(10):967–70. . 724298 10.1203/00006450-197810000-00003

[28] Gwirtsman HE, Kaye WH, George DT, Carosella NW, Greene RC, Jimerson DC. Hyperamylasemia and its relationship to binge-purge episodes: development of a clinically relevant laboratory test. J Clin Psychiatry. 1989;50(6):196–204. . 2470728

[29] Scheutzel P, Gerlach U. [Alpha-amylase isoenzymes in serum and saliva of patients with anorexia and bulimia nervosa]. Z Gastroenterol. 1991;29(7):339–45. . 1950041

[30] Kronvall P, Fahy TA, Isaksson A, Theander S. The clinical relevance of salivary amylase monitoring in bulimia nervosa. Biological Psychiatry. 1992;32(2):156–63. 1384726 10.1016/0006-3223(92)90018-u

[31] Sramek JJ, Cutler NR, Hurley DJ, Seifert RD. The utility of salivary amylase as an evaluation of M3 muscarinic agonist activity in Alzheimer's disease. Prog Neuropsychopharmacol Biol Psychiatry. 1995;19(1):85–91. . 7535938 10.1016/0278-5846(94)00107-s

